# Finding connection “while everything is going to crap”: experiences in Recovery Colleges during the COVID-19 pandemic

**DOI:** 10.1186/s40900-023-00489-4

**Published:** 2023-09-07

**Authors:** Holly Harris, Rowen Shier, Georgia Black, Anna Di Giandomenico, Elizabeth Lin, Gail Bellissimo, Jordana Rovet, Sam Gruszecki, Sophie Soklaridis

**Affiliations:** Department of Education, Centre for Addiction and Mental Health, 1025 Queen St. West, Toronto, ON M6J 1H1 Canada

**Keywords:** Recovery College, Pandemic, Mental health, Co-design, Participatory action research, Inclusion, Personal recovery, COVID-19

## Abstract

**Background:**

Recovery Colleges (RCs) are mental health and well-being education centres where people come together and learn skills that support their wellness. Co-production, co-learning and transformative education are fundamental to RCs. People with lived experience are recognized as experts who partner with health professionals in the design and actualization of educational programming. The pandemic has changed how RCs operate by necessitating a shift from in-person to virtual offerings. Given the relational ethos of RCs, it is important to explore how the experiences of RC members and communities were impacted during this time. To date, there has been limited scholarship on this topic.

**Methods:**

In this exploratory phase of a larger project, we used participatory action research to interview people who were accessing, volunteering and/or working in RCs across Canada. Semi-structured interviews were conducted with twenty-nine individuals who provided insights on what is important to them about RC programming.

**Results:**

Our study was conducted amid the COVID-19 pandemic. Accordingly, participants elucidated how their involvement in RCs was impacted by pandemic related restrictions. The results of this study demonstrate that RC programming is most effective when it: (1) is inclusive; (2) has a “good vibe”; and (3) equips people to live a fuller life.

**Conclusions:**

The pandemic, despite its challenges, has yielded insights into a possible evolution of the RC model that transcends the pandemic-context. In a time of great uncertainty, RCs served as safe spaces where people could redefine, pursue, maintain or recover wellness on their own terms.

**Supplementary Information:**

The online version contains supplementary material available at 10.1186/s40900-023-00489-4.

## Abstract (plain language summary)

**Background:**

Recovery Colleges (RCs) are mental health and well-being education centres where people come together and learn skills that support their personal recovery journeys.

**What did we want to know? What approach did we take?:**

In this phase of a larger project, we used a participatory action research approach to interview people who were accessing, volunteering and/or working in RCs across Canada. This research approach draws on the knowledge of all researchers and participants and places equal value on personal and professional experiences. Therefore, this study was created and shaped by, with, and for people who participate in RCs in partnership with academic researchers.

**What Did We Do?:**

Twenty-nine individuals shared what is important to them about RC programming. Our study took place during the COVID-19 pandemic during the pandemic-related restrictions such as social distancing.

**What did we learn?:**

The results of this study demonstrate that RC programming is most effective when it: (1) is inclusive; (2) has a “good vibe”; and (3) equips people to live a fuller life. The pandemic, despite its challenges, could inform an evolution of the RC model that lasts beyond the pandemic.

**Supplementary Information:**

The online version contains supplementary material available at 10.1186/s40900-023-00489-4.

## Introduction

Personal recovery is a nonlinear process of living a purposeful and meaningful life despite mental distress, challenges and adversity [1, 2]. Key components of personal recovery include connection, hope, a positive sense of identity, meaning and purpose [3]. In recent years, personal recovery-oriented practice has become mandated as a standard of mental health care in many countries [4]. Accordingly, drawing on their successful implementation in the United Kingdom, Recovery Colleges (RCs) have rapidly expanded to more countries, including Canada, to provide holistic approaches to support personal recovery [5]. RCs are mental health and well-being education centres where people come together and learn skills in support of their personal recovery journeys. RCs operate on an adult education model and host courses on a variety of topics including (but not limited to) understanding mental health and treatment options, vocational skills, life skills and recreation [6]. Those accessing RCs refer themselves to the program and select the courses that they feel are most aligned with their interests and needs. Offerings vary from 1-hour workshops to 10 weekly 2-hour sessions, with the majority of courses being two to 3-hour weekly sessions over 4 to 6 weeks. Central to the RC philosophy are the concepts of peer support, co-production and co-learning. In co-production, people accessing RCs are recognized and situated as experts who collaborate with people providing services to design, facilitate and actualize all components of the RC (e.g., course calendars, curriculum, logistics, course facilitation, strategic directions, governance) [7, 8]. By placing lived expertise on par with professional expertise, RCs open space to deconstruct hierarchical power structures, with members coming together to form a collaborative learning community [9, 10].

The purpose of RCs is to bolster personal recovery through a supportive and inclusive community. However, the restrictions placed on social gatherings during the pandemic posed challenges in meeting these goals and raised questions about the future of RC programming given in-person offerings were suspended. Research found that containment strategies, such as social distancing, contributed to an increase in anxiety, depression and mental distress [11]. Given the negative impact of the pandemic on mental health, personal recovery-oriented programming has become even more important [12].

The pandemic has changed how RCs operate by necessitating a shift from in-person to virtual offerings [13]. Given the relational ethos of RCs, it is important to explore how the experiences of RC members and communities were impacted during this time. To date, there has been limited scholarship on this topic. In this co-produced research project, we conducted interviews with people accessing, volunteering and/or working in RCs to understand what is important to them about RC programming. Because the study took place in the midst of the pandemic, an unexpected result was that it garnered insight into the impact of the pandemic on participants’ experiences. As COVID-19 related restrictions ease globally, RCs have adopted a variety of course delivery methods including hybrid, in-person and virtual. It is imperative that we critically reflect on the lessons learned through the pandemic and consider their applicability beyond this context.

### Terminology

To avoid stigmatizing language, which perpetuates damaging stereotypes, we have chosen terms that demonstrate our commitment to equity, diversity, inclusion and anti-discrimination. Consistent with language used by study participants and with the inclusive philosophy of the RC movement, we use action-oriented and person-first language throughout this paper (e.g., people accessing RCs).

## Methods

### Design

This qualitative exploratory study was part of a larger research project comprising two simultaneous phases that explored RC evaluation. The first phase involved the co-creation of a scoping review that examined how RCs were evaluated and whether they were co-produced with people accessing RCs [14, 15]. The second phase involved interviewing people who access, volunteer and/or work in RCs to explore two questions: (1) What do participants understand to be the most important elements of RCs for their personal recovery? and (2) How do we create evaluation measures that are relevant and impactful to people accessing RCs? This article focuses on the first question, with the latter covered separately [15]. While the impact of the pandemic was originally not a focus of our study, the timing of our interviews yielded rich information about how COVID-19 affected the experiences of people involved in Canadian RCs and what participants considered to be the key factors for supporting their personal recovery within the confines of the pandemic, and beyond.

We believe that co-producing research with members of the community of focus is necessary for ethical research and essential in promoting equity and inclusion. As such, the study used a participatory action research (PAR) methodology to engage people who attend or facilitate RCs, researchers and administrators as co-authors and co-investigators [14, 16]. PAR involves an iterative process of reflection, data collection and action to improve health, reduce health inequities and decentralize traditional research by involving those most affected [17, 18]. Underpinning PAR are principles of power sharing, leveraging strengths, empowerment, and honouring diverse perspectives and forms of knowledge [19]. It consists of three core elements: (1) active participation of researchers, knowledge users and study participants in the co-construction of knowledge; (2) promotion of self- and critical awareness and reflexivity leading to individual, collective or social change; and (3) building partnerships between researchers and knowledge users in all stages of research [18]. Consistent with the principles of PAR, we have included positionality statements from each author and invite readers to consider the perspectives represented, how they influenced the writing of this manuscript, as well as the voices that are missing (Table 1).

**Table 1:** Study authors’ positionality statements

Holly Harris	
I acknowledge the intersectional privilege/oppression that I experience on account of my identity. I am a white, middle-class, cisgender female with master’s-level education. I identify as someone who is neurodivergent and a consumer/survivor of the psychiatric system. I am employed by a tertiary mental health care facility as a research coordinator and have been involved in RCs as a peer support specialist, peer facilitator and research coordinator for 5 years. I leverage my lived experiences as a source of strength, resilience and expertise to highlight the voices of those who have been historically silenced. I acknowledge that my lived, academic and professional experiences influence the value I place on specific ideas and my interpretation of data	
Rowen Shier	
I am a white, cisgender female with a master’s degree in social sciences focusing on the intersection of policy and power relations. Although I am now positioned as middle class, I was raised in a lower income family. Additionally, I identify as someone with lived experience and have firsthand knowledge related to the inaccessibility of traditional mental health services. I am new to RC research and to work in the field of mental health and wellness more broadly. I acknowledge that my lens for engaging with this study has been shaped by my intersectional privileges and oppression and by the lived and learned experiences of my colleagues with whom I navigated this research	
Georgia Black	
I am a white, cisgender female who immigrated to Canada from Scotland in 2019. I have an undergraduate degree in psychology, and I have worked with populations that experience elevated rates of health inequity (including prison settings and homelessness services). I am currently employed as a research analyst in the education department of a large mental health hospital. My professional background is underpinned by my lived experience of accessing and navigating mental health services. I am not directly involved in the design, implementation and evaluation of RCs, but I am involved with research in this area. My approach to this research project is shaped by my lived and professional experience, including when co-creating meaning during interviews with participants and when interpreting the data	
Anna Di Giandomenico	
This project is my first experience with conducting research related to RCs. I have conducted patient-partnered research with Diabetes Action Canada and am an author on an academic publication related to that work. I have been a student in RCs for 5 years and have been a member of an RC course and programming committee for the last 3 years. I have a bachelor of arts in psychology as well as my early childhood educator certification. Leveraging my education and lived experiences as an RC student in this project has been a positive experience for me	
Elizabeth Lin	
I am a non-white, middle-class female with a doctoral degree and have dealt with barriers for non-whites in more than one country. I have been employed by a tertiary mental health care facility as a research scientist for over 30 years and have an extensive track record in traditional health services research using quantitative and large survey methods. I am new to RC research with this being the first project regarding RCs that I have been involved in. My interactions with individuals who have been involved in RCs has largely been with this team in writing manuscripts arising from this project and occasional administrative meetings where RC students, administrators, or facilitators have also attended. My role in this project included contributing specific expertise on conducting scoping reviews and editing manuscripts for scholarly journals. My perceptions are very much influenced by my upbringing and the cultural and social context that I grew up in and had to navigate to gain my education and my current occupation	
Gail Bellissimo	
I identify as a white middle-class, cisgender female. I acknowledge my social location and associated privileges, as well as experiences of stigma. I have spent 8 years engaging in research in the areas of chronic health, mental health, patient-oriented research and service user education. I have also been involved in the co-design of an RC for a large mental health care hospital. I seek opportunities to create inclusive and safe spaces by removing barriers to allow for capacity building, mentorship, education and peer support for the voices that are denied access due to discrimination and biases	
Jordana Rovet	
I recognize that my understanding of this subject is influenced by multiple intersecting aspects of my identity. I am a white settler, middle-class, cisgender female with a master’s degree in social work. I have spent the last 10 years working in the area of mental health and substance use, supporting projects led by people with lived experience. I am currently employed by a large mental health care hospital as a coordinator, where I am directly involved in the design, implementation and evaluation of an RC. My positionality informs the lens I bring to the data and the ways in which I interpret the experiences of others	
Sam Gruszecki	
I identify as a white cis-gendered middle-aged male. I work as a coordinator for an RC and am an employee of the organization that employs many of the people involved with this paper. This is part of my work. I also had collegial and community-based experience with most involved prior to the work starting. Some of my lived experience includes navigating anti-Semitism, neurodivergence, multiple diagnoses and experiences with poverty, as well as being the child of an immigrant, navigating services and a lack of post-secondary education. I have been involved in RC work, funded through major hospitals, as a peer support specialist, lead peer and coordinator since 2014. My experiences in research are relatively limited and I continue to learn along the way	
Sophie Soklaridis	
I am the daughter of Greek parents who immigrated to Canada. I grew up in Lourdes, Newfoundland, and Scarborough, Ontario. I hold assumptions and perspectives that are shaped by how I see/experience the world and how the world sees/experiences me. I currently work in an academic medical institution and recognize that it is a privileged site of knowledge production that has historically marginalized paradigms outside of the traditional biomedical model. I strongly believe in the importance of collaborating with lived-experience experts in research. My intent is to use my positional power to amplify their voices as experts with invaluable knowledge to contribute to the research process	

### Study participants and recruitment

Recruitment was facilitated by administrative personnel at six RC sites across Canada. A study information letter was circulated directing interested participants to contact the research analyst (GB) or a trainee. Table 2 lists inclusion and exclusion criteria. The consent process took place remotely via telephone or Webex (video conferencing software) [20].

**Table 2:** Inclusion and exclusion criteria

Inclusion criteria	Exclusion criteria
At least 18 years old	Under 18 years old
Currently accessing, volunteering and/or working at a Canadian RC	Not currently accessing, volunteering or working at a Canadian RC
Speaks and understands English	Does not speak or understand English
Has the capacity to consent to research participation	Does not have the capacity to consent to research participation

### Data collection

Consistent with PAR values, the qualitative interview guide (see Additional file 1) was designed to empower participants to share perspectives and experiences in their own words and contribute to the co-construction of knowledge between participant and interviewer [21]. Co-constructing knowledge entails iteratively building shared understanding and meaning with research participants [22]. Twenty-nine semi-structured interviews were conducted via Webex or telephone with people who access (*n* = 25), volunteer and/or work (*n* = 9) at Canadian RCs between December 2021 and June 2022. It is important to note that several participants held multiple roles in connection with RCs. Interviews were conducted by GB or a trainee and lasted approximately 60 min. Interviews involved open-ended questions related to participants’ involvement with RCs, perceived key program features, RC evaluation methods and suggestions for improved evaluation techniques. Participants received a $20 honorarium in the form of a gift card. All interviews were audio-recorded, transcribed and de-identified.

### Data analysis

Our team adopted an inductive approach to thematic analysis [23]. Data were analyzed iteratively and collaboratively as a team. Initially, team members independently coded two transcripts and met to compare findings and discuss thoughts and reflections. The codebook was then co-designed by incorporating feedback from all authors. Once consensus was achieved, the codebook was uploaded into Dedoose 9.0.46 software [24], where the team double-coded one additional transcript to establish interrater agreement. Researchers (HH, RS, GB, AD, GBel, JR, SG) coded another two or three transcripts each, implementing the reflexive practice of memoing to document emerging impressions and relationships within the data [25], which the coders then explored at weekly meetings. RS completed the initial coding of the remaining transcripts. Next, SS led the team in an iterative process in which codes and memos were grouped into high-level descriptive categories with example quotations from the transcripts. After ensuring that categories were inclusive and reflective of the entire data set, the team collectively refined categories into three central themes: (1) facilitating inclusion; (2) creating a “good vibe”; and (3) living a fuller life.

## Results

### Theme 1: facilitating inclusion

A central tenet of the RC philosophy involves offering inclusive and low-barrier access to wellness education. When participants described the notion of facilitating inclusion, the majority were referring to three related concepts: (1) physical dimensions of accessibility; (2) low financial barriers; and (3) “being with.”

#### Physical dimensions of accessibility

Due to the need for social distancing during the pandemic, RCs shifted from in-person offerings to video conferencing platforms. The majority of participants described how changes in physical aspects of RCs affected their experience of attending courses from an accessibility perspective. Some participants emphasized the importance of offering a virtual meeting space at a time when it was difficult to access their usual social networks:

P003: The pandemic happened, and even the connections I did have were kind of broken because I couldn’t meet anybody [...] So having these regular [RC] sessions where I was seeing the same people again and again and discussing the difficulties they were having in their lives really helped me feel less like there was no support system around me at all.

P011: Because it’s during COVID, [the RC] is like a life changer. You finally have something to do, and support your mental health, while everything is going to crap.

Many participants described how virtual courses facilitated accessibility, particularly in terms of managing social anxiety, reducing commute times and enabling attendees to balance personal obligations:

P008: A year ago, I was struggling with social anxiety. I really didn’t like going outside, seeing anybody. I was kind of stuck in the house. So, a lot of times I would hide myself behind the camera. After a while it was like, you know what, I don’t need to do this anymore. I’m safe in my house. So I opened up the camera [...] I was very comfortable.

Some participants spoke about the ability of virtual courses to broaden the reach of RCs:

P004: It has opened it up to a lot more people, and it has made it so people who maybe are anxious about joining a class can come on and have their camera off if they need to and still feel comfortable to be there. They can communicate through the chat.

However, one participant highlighted how virtual RC attendance is not accessible for people without internet access:

P018: Until recently, I didn’t have the internet or a device with a webcam on it to allow me to attend. I finally acquired said items, and now I’m able to attend.

Several participants also described how social connections were hindered when physical accessibility to programs was stopped or limited during the pandemic. In contrast to in-person RC offerings, virtual platforms make it more difficult to read and respond to visual social cues:

P026: But the in-person class, I feel that we have better communication. We can see each other’s gestures. We can connect with each other and other students.

#### Low financial barriers

Several participants described how free courses are a key component of RC accessibility and inclusivity. One participant explained that compared with other, often cost-prohibitive, mental health services, free RC courses gave them a more comfortable environment in which to communicate their thoughts and feelings:

P027: In an RC situation, we can talk for an hour. In a psychiatrist’s office, 15 minutes costs $1000 [...] You don’t express yourself. You don’t say what you want to say because these people are professionals. They will label you.

Other participants described virtual courses as an additional factor in reducing financial barriers:

P018: Because of my financial situation, I cannot afford transportation to X and back [...] Being able to do it online allows me to attend like normal, and it allows me to learn just like any other student.

P021: It’s convenient. I don’t have to go anywhere, which is good because gas is through the roof [...] With my kid too [...] I didn’t have to worry about having to get someone to look after her.

#### Being with

Several participants described being supported by and “being with” other RC members as experiences where they felt a profound sense of belonging. Specifically, several participants described the notion of “being with” as key to dismantling traditional teacher/student hierarchies:

P007: [T]he facilitators make you comfortable very quickly. They encourage you to participate. But again, there’s no pressure.

P024: They’re there by choice. They’re members in a collaborative relationship of recovery. Not students. We’re not teachers [...] I myself am not comfortable with the word “student.” I believe that creates a power dynamic [...] I’m asking you to share with me. I’m thanking you for it. You’re teaching me. That’s collaborative.

Participants also described how the people facilitating courses lay the foundation for inclusive, respectful and supportive group dynamics:

P018: [Facilitators] really understand me and what I’ve been through and what I’m currently going through. They make life more comfortable. They’re sort of somewhere between your best friend and family.

Participants repeatedly described how their peers’ empathetic attitudes contributed to the centrality of supportive connection in both their RC experiences and their individual processes of personal recovery:

P005: The participants were also very engaging, as well, and very supportive of each other. It’s like building a friendship or a trust. It takes time where you feel that it’s okay to talk about certain things [...] There’s no judgement. There’s acceptance.

Participants also benefited from an environment that responded to and supported their unique needs:

P022: The format is nicer to learn things in than a traditional class because you know that people [...] are understanding and accommodating with any kind of mental health or learning issue.

P029: I’m very happy that X has an RC; particularly, they have activities where people are allowed to speak their mother tongue.

### Theme 2: creating a “good vibe”

Several participants used the term “vibe” to describe the feeling or mood that was set by the people facilitating and other people participating in the RC. When participants described the vibe, the majority were referring to three related dimensions: (1) atmosphere; (2) attitude/approach; and (3) a sense of shared humanity in knowing that they were not alone.

#### Atmosphere

Several participants described how, despite not being able to meet in person, they still felt the “group vibe” over video conferencing. Although initially some participants worried that a virtual environment might make sharing their thoughts uncomfortable, these fears were not realized. Much of the ease of creating a story-sharing atmosphere was attributed to the people facilitating RC courses:

P026: The courses were virtual, but we can feel the positivity out of the computer when I see them [facilitators]. The way they explain the skills and information was so good and effective.

P021: I thought it would be uncomfortable [...] and I wouldn’t want to tell my stories. It wasn’t like that. The facilitators were very welcoming, very inviting and approachable online.

Those facilitating often shared their lived experience as a way to create an atmosphere conducive to open discussion and to deepen understanding of a particular topic. As this participant who is involved in RC facilitation explains:

P015: I think that when somebody leading the session can let their guard down and really open up as far as their lived experience and the things they have been through, you feel the energy in the room and *the vibe in the room* kind of settle down a bit. It feels good [...] I think when we’re able to share our stories it can make conversation much more valuable [...] It’s just about an energy and a *vibe*.

Because those facilitating courses created a “good vibe,” many participants described feeling at ease and relieved knowing that the atmosphere created would set the tone for others to act in an emotionally supportive manner. As this participant describes:

P005: It’s listening to people, and people listening to each other, and having the confidence and the support knowing that you can’t really say anything wrong. No one there is going to jump down your throat. It’s a support system.

#### Attitude/approach

Participants also described how the educational approach of the people facilitating promoted a supportive environment:

P026: I think it is more than the information [...] It’s the way they represent it for us. Because it’s important how we connect with them, how we feel about the information [...] But as a personal experience, I feel the difference between the way they represent this information.

Many participants described a strong sense of connection to other group members and a genuine desire to help one another during times of struggle:

P008: If somebody is having a bad day, immediately everybody starts typing, sending out hugs, sending out love, sending *good vibes.* It’s just such a nice feeling to have those people.

#### Sense of shared humanity

Participants described at length how the pandemic and mental distress were isolating experiences. The RC courses offered an opportunity to connect with other people. The majority of participants used words like “friendship,” “trust,” “non-judgmental” and “acceptance” to describe how they felt supported and engaged:

P012: What I really like about the RC is the community that we have. You can go to a group, and you know that people aren’t going to judge you because you’re living with mental illness.

P014: It’s really nice to connect with people who are going through similar issues [and] it came at a key time, because with the pandemic I’ve been feeling a lack of connection like many people. And it was just really good to talk, and just be in that environment.

Due to societal stigma associated with mental distress, for some participants, the RC was the only safe place in which they could come together and share their stories with people who understood them. When those facilitating opened the door to sharing stories, it created a trusting environment and let participants know that they were not alone.

P004: I find opening up and telling people [my story] [...] I see such a difference in how they open up. My story doesn’t have a happy ending. My story isn’t a good story. But I just see such a difference when people [...] It’s like all of a sudden they can trust me [...] they can relate to me. I see a light come on for them [...] they’re not alone.

Some participants thought that longer courses would help to build relationships. They described the RC as a supportive atmosphere, but explained that it took time to get used to the environment and to the program structure. Initially, course topics or the course structure could be triggering and “a bit uncomfortable,” but once participants “embraced the community,” it was a great experience.

P005: There’s a certain amount of trust, safety, etc. before you feel that you can open up. If it takes a couple of weeks, then you are kind of just starting to be a little bit more participatory, and then it’s like, “Oh well, it’s over.” Then you have to start building that relationship again with another group.

In addition, participants described how they can “feel the positive vibes” when they engage in RC programming. The trusting atmosphere and non-judgmental attitude and approach demonstrated by those accessing and facilitating created an affirming space that enabled participants to share experiences and that promoted a sense of belonging to a community:

P025: I really like how the RCs gave that freedom of “come as you are.”

P008: What I’ve enjoyed the most is the feeling of being in a community with other people who know what I’m going through. I like the fact that we support each other so well [...] I think it has a lot to do with the peer support specialists. They give off this vibe. They make you feel so welcome.

### Theme 3: Living a fuller life

When participants described the notion of what it means to live a fuller life, the majority were referring to three related dimensions: (1) having an opportunity to flourish; (2) learning with like-minded peers; and (3) living the change. Although this theme is not pandemic specific, it is important to highlight that RCs created spaces for people to work through challenges in pursuit of their goals at a time when many opportunities were limited due to pandemic restrictions.

#### Having an opportunity to flourish

Participants described the RC as a place that provided space to flourish. The supportive environment created in the RC showed participants a path toward (re) gaining a purpose in life:

P006: You are creating an environment where recovery and flourishing can take place. So the RC offers a different kind of environment. You’re creating an environment for people to achieve and to move forward. You can move towards your hopes and your dreams.

For many, the RC was a safe place to understand and process feelings such as anxiety, grief and suicidal ideation. Several participants credited participation in an RC as the reason they’re alive today. They were also clear that they felt this way because the RC was not a place for treatment or therapy. Therapy was important, but the clinical environment was distinctly and importantly different from RCs. For participants, the RC was more about learning how to live a fuller life:

P019: I think for some people they still need to do therapy or see a psychiatrist or do whatever. But there are a lot of people that are living a life that is not full. I think if they were to take part in some things like this [RC], they would kind of transform into a phoenix. They’d be able to fly and feel good about things again. I can’t really put into words how much my life has changed because I felt like I was nothing, and now I feel like somebody. I feel important.

#### Learning with “like-minded” peers

Some participants depicted the RCs’ peer-to-peer support of “like-minded” individuals as an important alternative to the traditional power dynamics that exist in mental health services. RCs provided a context for exploring individual autonomy:

P024: The clinicians, the doctors, the researchers, the methodologists, they see no problem with acronyms and certain words. But they alienate and stigmatize the marginalized populations. Period. That’s why there’s so much power and credit to things like peer support and RC. It’s like-minded individuals with lived experiences. The chances of a positive result, by inserting hope through the credibility of someone who has been there, is when you avoid as many of these stigmatic words as possible. The verbiage is very, very important. We’re not our diagnoses. We’re not students. We’re people who happen to teach and share with each other.

For some participants, the notion of living a fuller life included learning from, with and about fellow peers:

P012: I have found new ways to cope and maintain my recovery from both the instructors and also other group members. So, I have more tools in my recovery toolbox than I did when I ended up at the clinic.

Participants spoke at length about the importance of being around “like-minded” people and its contributions to learning:

P005: I found the women’s group, or the peer discussion, very beneficial as well. Because at the time, with COVID and things [...] there is not a lot of contact with people. Especially with like-minded people.

P021: Although we were from different walks of life, we all shared the same issue, I guess. We were all there for the same reason. It was very positive and comfortable, so that is why I kept with it.

Several participants described how learning was multi-directional:

P015: Throughout the session, it is generally about stimulating discussion and stimulating learnings, both on their end [participants] and even our end [facilitators]. That is kind of what I love about the RC format—the idea that we’re all learning from each other, at the end of the day.

#### Living the change

RCs were seen as places where participants could engage in their recovery by transferring learned skills into everyday life. For example, during moments of crisis:

P018: It [RC] has allowed me to control all of that and realize life isn’t the catastrophe I anticipate it’s going to be. Take a step back, take a deep breath, do some relaxation exercises, come back, and look at my situation. The RC has given me step-by-step instructions on how to handle myself in those situations.

Participants described the educational opportunity of personalizing their involvement as a critically important aspect of autonomy through self-guided learning, self-assessment and personal growth:

P001: They would send you the handout for the week, and you could review them. Just looking at your progress as well, through your own account.

This participant shared how they took what they learned to “live the change”:

P013: I’m making my own [notes and slide shows] to make [the content] more real, and I can put them in a binder or on my bulletin board. So, I can really actually live it. I can live the change.

## Discussion

Given the ongoing impact of the COVID-19 pandemic, it is important to understand how changes to programming affect the experiences of people involved with RCs. Our study showed that RC programming is most impactful when it is inclusive, creates a “good vibe” and equips people to live a fuller life. The shift to providing virtual opportunities has ushered in a new and evolved RC model.

While the transition to online offerings ensured continuity of programming during the pandemic, the unique benefits of online courses seem to stretch beyond that context. As participants described, online offerings facilitate inclusion for people who are uncomfortable with in-person environments, live remotely, have burdensome transportation costs or have domestic responsibilities. However, participants also missed certain aspects of in-person RCs, such as opportunities for meaningful connection and accessibility for those without digital access. These findings suggest an appetite for a hybrid model that allows people to engage with RCs in different ways. This approach is consistent with the RC philosophy of maximizing opportunities for choice and honoring the right to self-determination. Overall, the pandemic and associated changes have yielded insights into how RCs can broaden their reach and deepen their commitment to accessibility beyond the pandemic context.

Despite some initial reservations about transitioning from an in-person to a virtual format, several participants described how video conferencing was able to maintain the “group vibe.” They noted that those facilitating were key to creating an atmosphere, attitude, approach and closeness conducive to sharing stories in a virtual space. This observation illustrates the importance of digital compassion [26]. Digital compassion is the ability to remain present and responsive to the needs of others in a virtual environment. Compassionate communication involves reading and responding to visual social and emotional cues that often do not translate digitally. This means that flexibility and creativity are required to demonstrate compassion in a virtual environment. Compassion is central to the personal recovery phenomenon and finding ways to foster compassion in a virtual environment is essential to the RC model [27].

RCs are transformational spaces that support people in reimagining possibilities for their future and for living fuller lives. In a time of great uncertainty, RCs served as safe spaces where people could redefine, pursue, maintain or recover wellness on their own terms. Consistent with the personal recovery philosophy, participants noted that RCs helped them pursue and realize their purpose and potential. With the increased stressors and prevalence of mental distress that the pandemic generated [12], people accessing RCs had opportunities to gain skills to help them be resilient and flourish in the face of challenges and adversity. Ultimately, RCs offered space for people to work toward and achieve their goals in a time of great uncertainty.

### Limitations

Our original interview questions were not designed to seek information about participants’ pandemic-related experiences. The questions were broad and designed to elicit participants’ experiences in RCs in general. Participants related their experience with RCs during the pandemic organically and in conjunction with the researcher as the interviews progressed. Additionally, we did not collect nuanced demographic data because it is challenging to collect this type of data when the purpose of the interview is to build rapport and ask open-ended questions. Although collecting demographic data is not required in qualitative research, we recognize that collecting this information could have been useful in describing our sample. Finally, study participation was limited to people who could participate virtually given geographical barriers and physical distancing restrictions. People with no means to access technology or without the comfort and full ability to use technology were likely unable to participate in our study.

## Conclusion

This study elucidates how the imposed changes to RC operations during the pandemic can promote an evolution of the RC philosophy to further the reach and impact of programming on the personal recovery journeys of those who access RCs. The pandemic demonstrated that the RC model and RC communities are adaptable and resilient in the face of adversity. The insights gathered through our interviews can be used to inform quality improvement initiatives and processes to further prioritize the outcomes that are most important to RC community members.

In light of the current findings, future community-engaged research should examine how to evaluate and subsequently bolster the aspects of RCs that people accessing, volunteering and/or working at RCs have deemed to be most important in a way that they consider relevant and impactful. RCs should also consider how to leverage insights from the pandemic to deepen their commitment to inclusion and impact by offering multiple means of engaging with RCs. Future research could explore the impact of hybrid RC offerings and how virtual technology might shape and influence recovery-oriented educational initiatives within RCs. Given how essential compassion is in recovery-oriented practice and education, it is important to explore opportunities for training those facilitating in RCs in the unique competencies they need to practice digital compassion, such as communication skills, self-reflection and reflexivity [26]–[28]. In conclusion, the pandemic has offered unique insights into how the RC model can evolve to best reflect the things that matter to the people most closely affected by the programming.

### Supplementary Information

**Additional file 1:** Interview guide topics.

## Data Availability

Aggregate de-identified data is available upon request from the lead author.

## Abbreviations

RC: Recovery College
PAR: Participatory action research
