# Supplementary material for: Finding connection “while everything is going to crap”: experiences in Recovery Colleges during the COVID-19 pandemic
Source: Res Involv Engagem. 2023 Sep 7;9:77. doi: 10.1186/s40900-023-00489-4 (PMC10485942; doi:10.1186/s40900-023-00489-4)
Supplement: Supplementary file 1 — Additional file 1: Interview guide topics. [file 40900_2023_489_MOESM1_ESM.docx]

APPENDIX 1: Interview guide topics

|  | Topic area | Sample question |
| --- | --- | --- |
| 1 | Overall experience of Recovery College | How would you describe your experience with Recovery Colleges? |
| 2 | Experience of other services compared with Recovery College | How would you compare your experiences with Recovery Colleges with your experience of other services? |
| 3 | Positives of Recovery College | What does the Recovery College do well? |
| 4 | Areas of improvement for Recovery College | What could the Recovery College improve on? |
| 5 | Evaluating Recovery College: content of evaluation | Following explanation of evaluation/developing evaluation framework:  What parts of the Recovery College might be important to evaluate? |
| 6 | Evaluating Recovery College: methods of evaluation | Following on from discussion about what might be important to evaluate:  What would be the best way to go about capturing information for an evaluation of the Recovery College? (example prompt: different types of methods e.g., interviews/surveys/town halls) |
